# Sexual, bladder and bowel problems in people with spinal cord injury in rural KwaZulu-Natal, South Africa

**DOI:** 10.4102/ajod.v14i0.1480

**Published:** 2025-04-30

**Authors:** Lauren Tomes, Sonti Pilusa

**Affiliations:** 1 Department of Physiotherapy, Faculty of Health Sciences, University of the Witwatersrand, Johannesburg, South Africa

**Keywords:** spinal cord injury, bowel problems, bladder problems, sexual problems, long-term care needs

## Abstract

**Background:**

Spinal cord injury (SCI) is a life-changing experience that comes with multiple health challenges such as bowel, bladder and sexual health problems. Studies on the experiences of people with SCI based in rural South Africa are scarce.

**Objectives:**

This study aimed to explore the experience and long-term care needs related to sexual, bowel and bladder problems in people with SCI in a rural setting.

**Method:**

An exploratory qualitative design was employed. Semi-structured interviews were conducted with people with SCI living in rural KwaZulu-Natal. The interviews were transcribed verbatim and coded. The content analysis steps were followed to identify categories and themes.

**Results:**

A total of 12 individuals with SCI were interviewed. Frustration was the main theme that emerged with three sub-themes: types, management and effects of sexual, and bladder and bowel problems on individuals with SCI. The expressed long-term care needs were medication specific to SCI conditions, health information on secondary health conditions and prevention care, and resources such as nappies and quality catheters.

**Conclusion:**

The findings confirm that secondary health conditions such as bowel, bladder and sexual health problems affect the well-being of people with SCI in rural settings. Prevention care is urgently needed.

**Contribution:**

Patient education information on bowel, bladder and sexual health problems, and access to medication is imperative to support self-management practice.

## Introduction

Spinal cord injury (SCI) is a traumatic experience resulting in long-term disability and vulnerability to developing secondary health conditions (SHCs) (Adriaansen et al. 2016). Secondary health conditions such as pain, pressure sores, and sexual, bowel and bladder problems are common health problems in people with SCI (Pilusa, Myezwa & Potterton 2021a; Strøm et al. 2022). One SHCs cluster that tends to occur because of sympathetic and parasympathetic dysfunction related to the SCI, is the combination of bowel, bladder and sexual health problems (Park et al. 2017). A multi-country survey on SHCs found a very high prevalence of bowel, bladder and sexual health problems (70.8%, 62% and 71.3%, respectively) (Strøm et al. 2022). Unfortunately, bowel, bladder and sexual problems tend to decrease quality of life and well-being (Mashola & Mothabeng 2019; Park et al. 2017).

General prevention care for people with SCI needs strengthening. In general, prevention services for patients with SCI are not prioritised (Lofters et al. 2019). Fuseine et al. (2018) reported barriers and facilitators in SCI care in Ghana. Some barriers included health professional attitudes and poor access to the health education needed. Studies in South Africa have also reported contextual factors influencing SCI care and prevention care for SHCs (Bezuidenhout et al. 2023). Environmental factors included social attitudes and support, employment opportunities, inaccessible public transport, health professionals’ lack of knowledge of SCI and SHCs, access to medication and assistive devices (Bezuidenhout et al. 2023; Pilusa, Myezwa & Potterton 2021b). Personal factors that influence the prevention of SHCs include lifestyle behaviour choices, owning an assistive device and patients owning their health (Pilusa, Myezwa & Potterton 2021c). These factors may be worse in a rural setting where resources are limited; therefore, such studies focussing on rural-based patients with SCI are recommended.

Literature suggests that managing SHCs is not easy for people with SCI (Fuseini, Aniteye & Alhassan 2019; Guilcher et al. 2013). Poor access to timely health information, lack of follow-up care, a reactive health system that only focusses on treatment instead of prevention care and a shortage of medicines make SHCs challenging (Guilcher & Jaglal 2011; Pilusa, Myezwa & Potterton 2021d). Studies on SHCs have been conducted in urban settings in South Africa and there is a need to understand the experience and care needs of people with SCI in rural areas. This study aimed to explore the experience and long-term care needs related to sexual, bowel and bladder problems in rural-based people with SCI.

## Research methods and design

This study was conducted as part of L.T.’s MSc research, which sought to understand the lived experiences of people with SCI and their long-term care needs related to bladder, bowel and sexual problems. Two of the objectives of the study included an exploration of the lived experience of bowel, bladder and sexual problems in people with SCI and the identification of long-term care needs related to bowel, bladder and sexual problems in people with SCI.

### Study setting

The study was based in a district hospital in rural uMkhanyakude district, KwaZulu-Natal. uMkhanyakude district is one of the most rural areas in South Africa marked by a high unemployment rate, inaccessible roads and limited access to essential amenities. The district hospital renders inpatient and outpatient rehabilitation services that include physiotherapy, occupational therapy, dietetics and speech therapy. In addition, the rehabilitation unit has a partnership with a community-based non-profit organisation (NPO) for people with various disabilities including SCI. There are 53 people with SCI in the catchment area. The NPO has seven peer supporters who live with a disability and they play a critical role in disability care in the hospital. The peer supporters offer support and health education to people with disability in their care journey. When visiting individuals with disabilities, the peer supporters are accompanied by a qualified occupational therapist and physiotherapist to ensure holistic disability management.

### Study participants

Peer supporters affiliated with the NPO helped to identify and recruit community-dwelling adults with SCI irrespective of the level, duration and severity of the SCI, and those above 18 years of age. Purposive sampling was used to recruit people with SCI in the community and in the hospital. Only one potential participant was admitted for pressure sore; therefore, the interview was conducted in the hospital.

### Data collection

All the interviews were conducted in the participant’s preferred language, taking approximately 30 min to an hour. We audio-recorded the interviews and took notes on the participants’ impressions and the discussion. Discussions between the researchers were conducted after the interviews. The interviews were conducted from 19 to 22 April 2022. Data collection stopped when no new information emerged.

### Data analysis

All the interviews were transcribed and then translated into English. The researchers read transcripts making sure the translation was accurate. MAXQDA software version 2018.2 was used for data analysis. We followed the following steps outlined by Erlingsson and Brysiewicz (2017):

- Read and re-read to become familiar with the content of the transcripts.
- The researchers coded the transcripts inductively separately.
- We categorised similar codes and identified the overarching themes.

### Rigour

The following activities were conducted to ensure the trustworthiness of the process and the study findings: we have explained in detail the research process and setting, all the participants were purposively selected and we captured personal reflections and observations. All interviews were audio-recorded. The researchers held debriefing sessions throughout the research process.

### Reflexivity

Both researchers are physiotherapists with an avid interest in public health and disability management. The author S.P. conducted research on SCI and SHCs in an urban area. Our approach to care has always been to understand the unmet care needs to inform care and relevant intervention strategies. Not many physiotherapists focus on sexual, bowel and bladder problems; but from our observation, these three SHCs are neglected in clinical rehabilitation.

### Ethical considerations

Ethical approval to conduct this study was obtained from the University of the Witwatersrand Human Research Ethics Committee (No. M210813). The South African National Health Research Database (No. KZ_202201_018), the district hospital and the NPO granted permission to conduct the study. The study aim and process were explained in detail in IsiZulu to all the individuals with SCI. Written consent and permission to audio record the interview were granted before conducting the interviews. Semi-structured face-to-face interviews using an interview guide with open-ended questions were conducted in the preferred setting stated by the participant. Nine interviews were conducted at the participants’ homes, two at the hospital because one participant was admitted for a pressure sore and the other one was in the physiotherapy department. The questions included in the interview guide with probes to facilitate the interview are listed in Table 1.

**TABLE 1:** Interview Guide with open-ended questions for face-to-face interviews.

Variable	Questions
Life with a disability (SCI)	Can you tell me about yourself … and your disability? Probes Can you tell me about your experience living with spinal cord injury? How and when did the disability occur? How did it change your life?
Bowel problems	Can you tell me about your bowel function and the problems that you may sometimes experience? Probes Can you share your experience with bowel problems in your life? Can you share with me how this problem affects your life? How do you manage this problem? what do you need to better manage bowel problems?
Bladder problems	Can you tell me about your bladder function? Probes Is it similar to your bowel function experience? Can you share your experience with bladder problems in your life? Can you share with me how this problem affects your life? How do you manage this problem? What do you need to better manage bladder problems?
Sexual problems	Some people with SCI experience sexual problems. Can you share with me about the sexual problems you have experienced since SCI? Probes Can you share with me how sexual problems affect your life and relationship? How do you manage this problem? What do you need to better manage sexual problems?
Suggestions	How can we improve care for these problems as healthcare professionals?

SCI, spinal cord injury.

## Results

Out of 53 SCI people in the catchment area, we only managed to interview 12 people with SCI. The majority were men. All the participants experienced SHCs: sexual, bowel and bladder problems, pain, spasms, pressure sores and contractures. Table 2 shows the participants’ demographic profile.

**TABLE 2:** The participants’ demographic and injury profile.

Participants	1	2	3	4	5	6	7	8	9	10	11	12
Age (years)	28	41	51	34	57	30	55	51	55	40	46	38
Gender	M	M	M	M	F	M	M	M	F	M	M	M
Date of injury (years)	3	19	10	9	20	3	33	18	20	13	5	7
Level of injury	T8	L4	L5	L3	L1	L3	L12	L2	T12	C4	T12	T12
Type of injury	Paraplegic Incomplete	Paraplegic Complete	Paraplegic Incomplete	Paraplegic Incomplete	Paraplegic Incomplete	Paraplegic Incomplete	Paraplegic Incomplete	Paraplegic Incomplete	Paraplegic Incomplete	Quadriplegic Complete	Paraplegic Incomplete	Paraplegic Incomplete
Cause of injury	Traumatic Stabbed	Traumatic Gunshot	Non-traumatic TB spine	Traumatic Gunshot	Non-traumatic TB spine	Traumatic Stabbed	Traumatic Gunshot	Traumatic Gunshot	Non-traumatic TB spine	Traumatic MVA	Traumatic Stabbed	Non-traumatic TB spine
Education level	High School Grade 11	High School Grade 10	No Schooling	High School Grade 11	No Schooling	Matric and post matric computer literacy	Primary School Grade 4	High School Grade 10	Primary School Grade 4	No Schooling	High School Grade 10	Matric
Marital status	Unmarried	Married	Unmarried	Unmarried	Unmarried	Unmarried	Unmarried	Unmarried	Unmarried	Married	Unmarried	Unmarried
Employment	Unemployed	Employed	Unemployed	Unemployed	Unemployed	Unemployed	Unemployed	Unemployed	Unemployed	Self employed	Unemployed	Unemployed
Secondary health conditions	Pain	Pain	Pain	Pressure sore	Pain	Pain	Pain	Pain	Pressure sore	Pain	Pain	Pain
	Spasms	Pressure sore	Spasms	Spasms	-	Pressure sore	Spasms	-	Spasms	Autonomic dysreflexia	Spasms	-
	Flaccid bladder	-	Bowel problems	Bowel problems	-	Contracture	-	-	Contracture	Fits	Bowel problems	-
	-	-	Bladder problems	-	-	-	-	-	-	Pressure sore	-	-
	-	-	Contractures	-	-	-	-	-	-	-	-	-

The theme related to the experience of sexual, bladder and bowel problems was ‘frustrating’.

The participants expressed a sense of frustration brought up by the lack of control over bladder and bowel problems:

‘I fail to control urine because when it pours esssh I cannot control it’. I don’t know how I can explain it because I really don’t have control over it.’ (P6, Male, 30 yrs, L3)

‘The bladder problem is very frustrating because I wake up at night being pressed with urine but end up fighting with my bladder for a very long time for it to release urine.’ (P1, Male, 28yrs, T8)

Similar sentiments were highlighted related to sexual problems. Although the participants engaged in sexual activity the sense of dissatisfaction with their sexual life left them feeling frustrated:

‘I often practice oral sex just to satisfy my wife, but I do not perform it daily for obvious reasons that there is no satisfaction on my side, and it does not make me feel happy.’ (P3, Male, 51yrs, L5)

‘I won’t lie, it (sex) is not good. I do it (sex), but I don’t get satisfied. Sometimes a woman can visit me expecting to have sex, but it just won’t happen. It is very bad.’ (P12, Male, 38yrs, T12)

Related sub-themes are presented in Table 3.

**TABLE 3:** Theme and sub-themes.

Theme	Sub-themes
Experiencing bowel, bladder and sexual problems is ‘frustrating’	Types of sexual, bladder and bowel problems experienced
	Management strategies used
	Effects of sexual, bladder and bowel problems on well-being

### Types of bladder and bowel problems

Table 4 outlines the types of bladder and bowel problems mentioned by the participants.

**TABLE 4:** Types of bladder and bowel problems.

Variable	Participant feedback
**Bladder problems**	
Urinary incontinence	‘I cannot control my urination but sometimes, I can control it. Like today, it was not very difficult but sometimes it gets difficult.’ (P5, Female, 57 yrs, L1)
Urinary tract infections	‘Sometimes I experience dark urine with bad smell even though I drink a lot of water, in that case, I go to see a doctor.’ (P1, Male, 28 yrs, T8)
Blood in urine	‘The blood in the urine has been coming out for a long time now.’ (P11, Male, 46 yrs, T12)
Pain in the bladder and when urinating	‘My bladder hurts a lot; I feel sharp pain and that makes me visit the hospital regularly.’ (P3, Male, 51 yrs, L5)
Urine retention	‘There are times when urine comes out fine because I use the urinary catheter to draw urine. After drawing urine there is more urine that pours just after drawing it. Just after removing the catheter, I feel the heat and more urine flows out.’ (P2, Male, 41yrs, L4)
**Bowel problems**	
Incontinence	‘I cannot feel it when I am pressed with stool. If I feel something it is a little feeling and I feel it very late by the time I must poo immediately, by that time you cannot rush to the toilet.’ (P1, Male, 28 yrs, T8)
Constipation	‘Sometimes I go to the toilet in a satisfactory way, but sometimes I spend the whole week without going to the toilet.’ (P2, Male, 41 yrs, L4)
Heartburn	‘I get heartburn when I eat any kind of food. I feel like food does not get digested correctly and that leads to constipation or hard stool constipation.’ (P4, Male, 34 yrs, L3)
Diarrhoea	‘I suffer from diarrhoea most of the time, at the worst diarrhoea almost takes all three weeks of the month leaving me with only one week of relief to recover.’ (P11, Male, 46 yrs, T12)

Mostly men stated the types of sexual problems (Table 5) they experienced: erectile dysfunction, poor sexual performance and the lack of sexual desire.

**TABLE 5:** Types of sexual problems.

Sexual health problems	Participant feedback
A lack of sexual desire	‘A few months after my injury I was sexually active, and my sexual feelings were in a good condition with a sexual appetite. From time to time there were changes in my sexual feelings which resulted in the loss of sexual appetite.’ (P2, Male, 41 yrs, L4) ‘Erectile dysfunction and low sex drive is another contributing factor for not being 100% fit for sexual intercourse.’ (P12, Male, 38 yrs, T12)
No enjoyment	‘I won’t lie, it is not good. I am weak now. Feel it happens that it takes long but a little bit, I do it, but I don’t get satisfied.’ (P11, Male, 46 yrs, T12)
Poor sexual performance	‘The challenge that I experience is that I cannot perform well.’ (P12, Male, 38 yrs, T12)
Erectile dysfunction	‘I get frustrated when my heart and mind think of sex, but I cannot have an erection since I do not have sexual feelings.’ (P2, Male, 41 yrs, L4)
Painful sex	‘This (healed pressure sore) pain also affects my penis; my penis gets the erection when I get close to my partner, but I also feel the pain on my penis, and I lose interest …’ (P2, Male, 41 yrs, L4)

### Management of bladder, bowel and sexual problems

**FIGURE 1:**
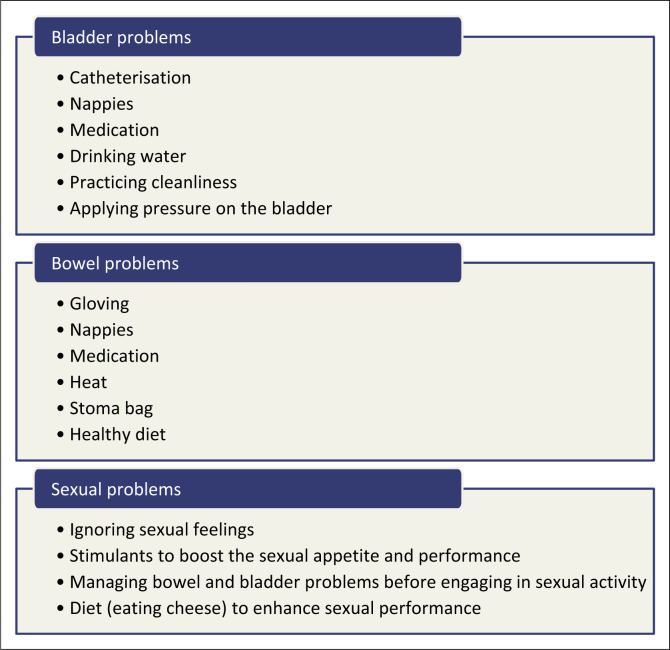
Management strategies.

Various strategies were used to manage sexual, bladder and bowel problems. Figure 1 outlines the management strategies for sexual, bladder and bowel problems.

### Effects on well-being

The effects of sexual, bowel and bladder problems were felt in different life domains: mental health, physical health, social lives, time and finances.

#### Mental health

The male participants stated that they had a lot of stress around their sexual life and inability to satisfy their partner sexually:

‘I feel stressed about the fact that I cannot have sexual intercourse with my wife to satisfy her sexual need … ’ (P2, Male, 41 yrs, L4)

Some men felt inhuman because they could not satisfy their partners’ sexual needs:

‘This stresses me a lot because I do not see myself as a complete man, for obvious reasons that I cannot satisfy my partner in bed and fulfil her sexual desire.’ (P11, Male, 46 yrs, T12)

Yet, one participant proved his manhood by having children even though he did not enjoy sex:

‘I got injured before having children, I got all my children after the spinal cord injury. That made me prove that my manhood exists.’ (P1, Male, 28 yrs, T8)

Fear was a common feeling among the participants. Some participants were afraid to lose their partners:

‘Sexual problems led to me losing hope in relationships and realising that there is no need for a relationship because we won’t do anything (*sex*) with a person I will fall in love with.’ (P1, Male, 28 yrs, T8)

‘I’m afraid that my woman will leave me for another man with sexual appetite, who will satisfy her sexual needs and make her feel happy.’ (Laughing …) (P11, Male, 46 yrs, T12).

Fear of leaking in public was evident in some participants:

‘When I’m around people I stay in fear that urine might just flow’, leading to isolation ‘Ahhh the problem of urine bothers me the most when I am around people. Sometimes, I isolate myself from people because I might wet my pants in their presence’ (P6, Male, 30 yrs, L3).

#### Physical health

Pain, pressure sores and contractures affected sexual health. The presence of pain from healed pressure sores, stiff muscles and joints caused discomfort during sexual activity, affecting positioning and lowering interest in sexual activity as reported by some participants:

‘I cannot lie for a long time on the side where I had a pressure sore because it gets painful. This pain lowers my sexual appetite, it hurts me because I can see that my partner is not sexually satisfied.’ (P2, Male, 41 yrs, L4)

‘Ever since my disability, I never had sex because my spinal cord area and legs are stiff, which makes it impossible for me to have sexual intercourse. My knees and muscles are also stiff and that limits the chance to try having sexual intercourse.’ (P3, Male, 51 yrs, L5)

The presence of a pressure sores made some participants decide to put aside their sex life:

‘Since I was injured and developed pressure wounds, I realised I would not involve myself in any sexual activities. It was early 2015 when I saw that I had pressure sores, I just chose to put aside my sex life.’ (P4, Male, 34 yrs, L3)

The presence of urinary incontinence also affected engagement in sexual intercourse as expressed by the participants:

‘During foreplay urine just flows.’ (P12, Male, 38 yrs, T12)

#### Social lives

The participants highlighted how their social lives were limited by the presence of sexual, bowel and bladder problems. Inability to satisfy partners sexually led to losing intimate relationships:

‘My previous sexual relationship ended because I could not satisfy my partner sexually, so we decided to go our separate ways’ (P6, Male, 30 yrs, L3).

Using public transport was difficult for most participants:

‘The flow of urine while you are trying to get into a car, your trip just gets interrupted. You will wet your pants. This thing affects my life.’ (P11, Male, 46 yrs, T12)

‘My bowel condition restricts me from using public transport because most drivers often assume that a person in a wheelchair might poo in their cars.’ (P12, Male, 38 yrs, T12)

#### Management takes time

The participants expressed how bladder and bowel management disrupted their lives because the process took too long. For example, few participants stated:

‘I wait for five minutes for urine to come out, but at that time I will be in a rush or need to go somewhere.’ (P1, Male, 28 yrs, T8)

‘When I visit the toilet, I am forced to wait for a long time before pooing because the stools are hard.’ (P12, Male, 38 yrs, T12)

### Long-term care needs

**FIGURE 2:**
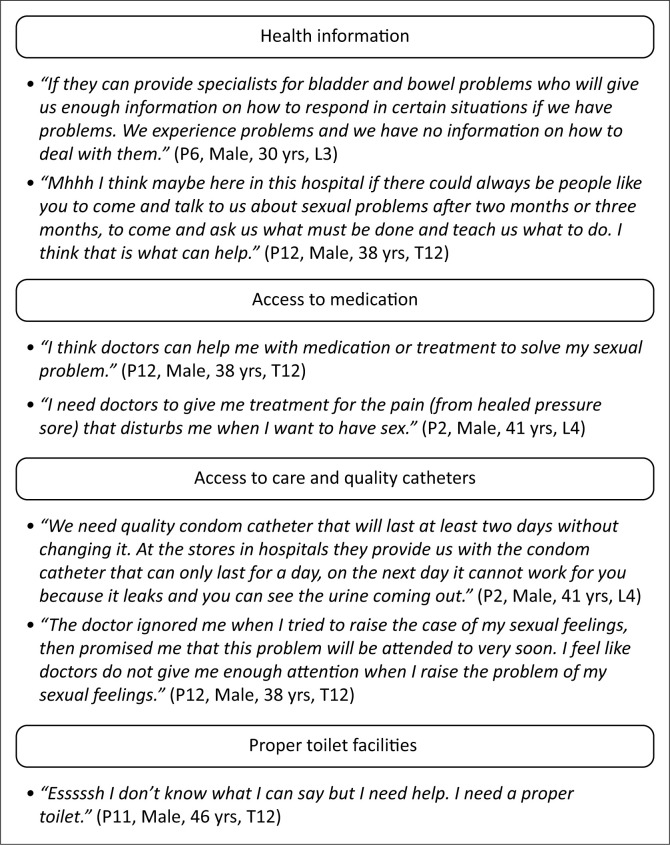
Long-term care needs related to sexual, bowel and bladder problems.

The participants stated that they needed more health information and access to medication such as Dulcolax, pain medication, sex stimulants and quality catheters. Figure 2 outlines the long-term needs related to sexual, bowel and bladder problems.

## Discussion

This is the first study on sexual, bladder and bowel problems in people with SCI based in rural South Africa. The participants expressed feelings of ‘frustration’ related to sexual, bladder and bowel problems. The presence of sexual, bladder and bowel problems can be disruptive and affect the quality of life. A sense of frustration and lack of control over long-term disability and resulting health conditions is common among people living with SCI (Callaway et al. 2015; Fuseini et al. 2019). Thus, long-term care support is needed to ensure challenges in self-care are identified and addressed.

As previously reported, the participants experienced a range of SHCs and co-occurrence of SHCs including sexual, bowel and bladder problems (Brinkhof et al. 2016; Park et al. 2017). Clustering or co-occurrence of SHCs such as pain, pressure sores and contractures can also make managing sexual, bowel and bladder problems challenging. When developing SHCs prevention and management interventions, it is essential to be holistic and consider interventions that address multiple SHCs (Dejong & Groah 2015).

It was good to hear that the study participants managed bowel and bladder problems using various strategies in line with SCI care guidelines (Sezer 2015). However, most participants used nappies when travelling or at night to avoid leakage. Nappies are costly and can be a risk for pressure sores. Support to ensure low-risk strategies for bowel and bladder management must be explored to lessen the financial impact on the individual with SCI. This study confirmed the struggle people with SCI experience in SHCs management and prevention practices (Chang et al. 2017). Managing sexual problems is not easy, despite the environmental setting, making some men feel devalued and inhuman. Issues of sexuality, sexual health and intimacy are important to people with SCI, albeit neglected. Conversations on sexual health, intimacy and challenges experienced should be incorporated into SCI care. Patient information can include sexual health, positioning, safe sex practices, and human immunodeficiency virus (HIV)/acquired immunodeficiency syndrome (AIDS) given that it is prevalent in our country.

Similar to previous studies conducted in urban settings, sexual, bowel and bladder problems affected the participants’ well-being (Callaway et al. 2015; Pilusa, Myezwa & Potterton 2021e). The participants expressed emotions of worry and fear related to sexual, bowel and bladder problems. Although SCI tends to be seen as a physical disability, the mental health impact of the injury and the SHCs must not be neglected. Moreover, the fact that most of the participants were unemployed young men with low education status consequently affecting their employment prospects, this too can also affect their mental health status. There is a need to ensure that mental health and well-being are regularly assessed and addressed in the SCI population by a multidisciplinary team. We also found that relationships were affected by sexual problems and bowel and bladder problems. Social support and relationships play a major role in health and disability outcomes. Although the findings are similar to the studies based in urban areas, environmental factors in rural areas are worse. For example, the roads to the participants’ homes were inaccessible. The roads were rough steep terrain. Getting out of the yard to see a neighbour or travelling a long distance to the local clinic or hospital was challenging. Thus, individuals resorted to paying taxi drivers, who were not always willing to transport them, more money to pick them up from their houses. We could understand the isolation and the desperation because of the SCI. Furthermore, rural settings are marked by low education status and high unemployment rate, as indicated by the participants’ sociodemographic data.

Long-term care needs include health information, access to medication, proper toilets and quality catheters. It was surprising that access to disability friendly transport and assistive devices was not mentioned as this also affects access to care (Pilusa, Myezwa & Potterton 2021b). Empowering people with SCI is part of patient-centred care and is critical for individuals with long-term conditions (Hudon et al. 2012). Given the cultural diversity in South Africa and inequalities in literacy levels, patient information must be made easily accessible in local languages. The beauty of the NPO we worked with was the peer supporters who could communicate in the local language. Furthermore, access to medication and catheters was also highlighted as a necessary intervention (Pilusa, Myezwa & Potterton 2022). We are advocating for better care planning for people with SCI. Care programmes for people with disabilities must adopt long-term perspectives to care ensuring access to essential services, assistive devices and medicine without worsening the individual financial status.

## Conclusion

The findings showed that sexual, bowel, bladder problems are frustrating for people with SCI. This study calls on health professionals and policymakers to strengthen SCI care and management for bowel, bladder and sexual problems. Health information sessions can be developed to empower people with SCI. Future studies are recommended employing participatory approaches to develop prevention and management interventions for sexual, bowel and bladder problems.

### Limitations

The study cannot be generalised as it depicts the experiences of people with SCI in this context. We had few female participants, and they did not share a lot about their sexual experiences. This could be because of cultural beliefs around sexual health. Most of the time, women are deemed to be recipients and may not face the same frustrations as men. The study was also conducted in rural South Africa, findings in urban areas may differ as social and environmental circumstances may differ.

### Implications

Research: we need implementation research on the impact of peer supporters and their role in the health system.

Practice and policy: incorporate peer supporters in the health system to support people with disabilities from diagnosis and throughout the rehabilitation phase.
